# Rescinding evidence-based care and practices during the initial COVID-19 outbreak in the United States: a qualitative study of the experiences of lactation support providers

**DOI:** 10.3389/fpubh.2023.1197256

**Published:** 2023-08-10

**Authors:** Julie Grady, Ellie Mulpeter, Kajsa Brimdyr, Karin Cadwell

**Affiliations:** ^1^School of Nursing, Curry College, Milton, MA, United States; ^2^Academy of Lactation Policy and Practice, South Dennis, MA, United States; ^3^Healthy Children Project, Inc., Harwich, MA, United States

**Keywords:** breastfeeding, lactation, counselors, pandemic, COVID-19, maternal–infant separation, policy

## Abstract

**Background:**

The COVID-19 pandemic disrupted healthcare systems and services including along the childbearing continuum. The aim of this study was to explore the experiences and perceptions of professional lactation support providers who cared for breastfeeding families during the early months of the pandemic (March 2020 – August 2020) in the United States.

**Design/methods:**

We conducted a qualitative survey among active lactation support providers in the United States. Eligible participants spoke English, were Certified Lactation Counselors who maintained an active certification and who provided lactation care and services prior to and after the onset of the COVID-19 pandemic. Participants were recruited via email from the national database of Certified Lactation Counselors obtained from the national certification body. All ten Health and Human Service regions of the United States were included. Demographic data was collected on each respondent. Qualitative survey responses were analyzed thematically following the framework method.

**Findings:**

Six-hundred and seventy-four (674) Certified Lactation Counselors responded to the survey from June to July of 2022. Their responses fell within the overarching theme of rescinding evidence-based care and practices that had been in place prior to the pandemic. Affected care practices included the insertion of limits on access to care and insinuating stigma and bias based on COVID-19 status. Irregular appointment schedules and staffing shortages also affected care. Participants reported that separation of the mother and their infant became the norm. Decisions made by management seemed to be grounded in fear and uncertainty, rather than on the evidence-based principles that had been in place prior to the pandemic.

**Conclusion:**

A lack of coordination, consistency and support, along with fear of the unknown, troubled lactation support providers and impacted their ability to provide evidence-based care and to maintain access to care for all families. The findings of the survey and analysis underscore the importance of adequately preparing for future public health crises by determining how evidence-based care and practices can be preserved in emergent situations.

## 1. Introduction

The SARS-CoV-2 virus spread rapidly across the globe in 2019, causing the Coronavirus pandemic (COVID-19). By the end of 2022, over 665 million cases had been confirmed worldwide, along with 6.67 million confirmed deaths (1). In the United States (U.S.) a national emergency was declared on March 13, 2020, causing a wave of partial or full lockdowns in 45 states and the District of Columbia (2). Healthcare providers and essential workers were asked to continue working while others were admonished to remain at home.

During the initial months of the pandemic, the fear of being exposed to the virus, bringing the virus home to loved ones, and falling seriously ill created a chaotic environment in many workplaces, and the context of healthcare was changed as a result (3). Unlike non-essential worksites, hospitals and birthing centers did not have the option of turning away patients or closing their doors. Additionally, the combination of uncertainty of how the virus was transmitted and the lack of knowledge about the management of those sick with the virus led to questions about best practice for care delivery (4). Clearly defined work routines of healthcare providers became disarranged.

One category of healthcare providers who experienced this change was the Certified Lactation Counselor (CLC^®^). CLCs are professional lactation support providers (LSPs) who construct and maintain conditions that predispose mothers and babies to an uncomplicated breastfeeding experience through counseling, education and support. CLCs provide clinical management of complex breastfeeding situations by assessing, monitoring, evaluating and providing evidence-based interventions (5). In the U.S. there are over 23,000 active CLCs in all 50 states and unincorporated territories working toward meeting the 2030 Healthy People Goals for Breastfeeding (6). CLCs represent the largest cohort of LSPs in the U.S. Despite the fear and uncertainty that COVID-19 presented, babies continued to be born. According to the National Vital Statistics Report, there were 3,613,647 births in the U.S. in 2020 (7), approximately 301,137 births per month and nearly 75,284 births per week. However, evidence has demonstrated that the percentage of exclusively breastfed babies significantly decreased during the pandemic in hospital settings (8). This parallels the observed decrease of lactation support in the hospital and a potential lack of community support for breastfeeding following hospital discharge (9, 10).

Breastfeeding and the provision of human milk is recognized as a foundation of public health. Since their inception, the U.S. Healthy People goals for the nation have included the goal to improve breastfeeding initiation and duration rates and have named this target again for their 2030 goals (11). The Joint Commission’s Perinatal Care core measures include reducing the use of formula in the hospital following birth and increasing exclusive breastfeeding as a perinatal quality indicator (12). The public health advantages of breastfeeding as well as the evidence-based practices needed to meet the Healthy People and Joint Commission goals are acknowledged and widely circulated (13).

Initially, the sweeping unknowns of the pandemic led professional organizations, such as the American Academy of Pediatrics (AAP) and the Centers for Disease Control and Prevention (CDC), to recommend the discontinuation of evidence-based practices (14, 15) such as skin-to-skin care in the delivery room and rooming in – two practices that support breastfeeding initiation, duration, and continuation (16). Other evidence-based practices that support breastfeeding were also affected, including companion support during labor, continuity of care and the provision of breastfeeding assessment, evaluation and support (17). The discontinuation of these practices resulted in measurable adverse effects. The COVID Mothers’ Study utilized an anonymous survey to gather information on COVID-positive mother–infant dyads worldwide and found that infants who did not directly breastfeed, experience skin-to-skin care, or room-in within arms’ reach of the mother were significantly less likely to be exclusively breastfed in the first 3 months of life (18). While the CDC altered their position in October 2020 and declared breastfeeding, keeping mothers and babies together following birth and rooming in safe, new families were not educated about the safety and benefits of breastfeeding, even up to one year into the pandemic (19, 20).

While initial studies on breastfeeding at the start of the pandemic have focused on the decline of breastfeeding initiation rates and the lack of access to care experienced across economic and racial backgrounds, there have been few studies analyzing the unique experiences of LSPs, such as CLCs, during this time (21). The primary objective of the current study is to focus on the unique experiences of CLCs, one specialized member of the healthcare team, as the COVID-19 pandemic began. Further, this research specifically examines the impact of the disruptions of evidence-based care on the experiences of CLCs in both hospital and community-based settings. The secondary objective of the current study is to examine how access to care was disrupted during the initial months of the pandemic, when viewed through the lens of the lactation support provider.

## 2. Materials and methods

For this qualitative study, the authors recruited CLCs across the U.S. states and territories who worked as a LSP during the initial months of the COVID-19 pandemic. Participants were invited via email to consent to and participate in an online questionnaire developed by the research team. The retrospective survey was conducted between 22 June 2022 and 7 July 2022. Participants were asked about the impact of the pandemic on their work, including policy and procedural changes that were adopted, the impact on their role as a CLC and challenges they faced in the workplace during the first months of the pandemic. Nine-hundred nine (909) CLCs accessed the online questionnaire and gave their consent to participate in the study. Of those, six-hundred and seventy-four (674) (74.1%) respondents completed the questionnaire in its entirety. Participant socio-demographic data was collected from those who completed the questionnaire (Table 1). Qualitative survey responses were analyzed thematically following the framework method. The authors first read and coded the responses line by line to allow familiarization. The second step involved the generation of descriptive themes and initial organization of participant responses into meaningful groups. The third step was the generation of analytical themes – themes that go beyond the simple descriptions of responses. In all three of these steps, the authors sought to understand the lived experiences of the lactation providers surveyed and the relation of those experiences to the pandemic-induced changes in the U.S. healthcare system. In the qualitative survey, respondents who answered “yes” to the initial question of whether or not their workplace implemented restrictions, were then asked to describe the changes in those workplace policies and procedures in their own words. They were also asked to describe how their roles as CLCs changed during that time.

**Table 1:** Demographic characteristics of respondents.

	*N*	%
Gender (*n* = 674)		
Female	665	98.66
Male	5	0.74
Gender variant/non-conforming	2	0.30
Chose to not respond	2	0.30
Race/ethnicity (*n* = 674)		
American Indian or Alaska Native	11	1.63
Asian	15	2.23
Black or African American	59	8.75
Hispanic/Latinx	53	7.89
Native Hawaiian or other pacific islander	3	0.45
White or Caucasian	507	75.22
Chose to not respond	14	2.08
Other	12	1.78
Age range (*n* = 674)		
18–24 years of age	4	0.59
25–34 years of age	181	26.85
35–50 years of age	310	45.99
50 years of age or older	178	26.41
Chose to not respond	1	0.15
Highest level of education (*n* = 674)		
High school diploma or equivalent (GED)	44	6.53
Associate’s degree	121	17.95
Bachelor’s degree	319	47.33
Master’s degree	165	24.48
PhD	12	1.78
MD	13	1.93
Current occupation (*n* = 674)		
CLC	348	51.63
Nurse	298	44.21
Nutrition worker	80	11.87
Manager/coordinator	67	9.94
Health educator	89	13.20
IBCLC	40	5.93
Speech language pathologist/occupational therapist	26	3.86
Physician	10	1.48
Social worker	13	1.93
Family advocate	19	2.82
Doula	61	9.05
Midwife	13	1.93
Other	146	21.66
Credentials (*n* = 674)		
CLC	280	41.54
CLC, RN	303	44.96
CLC, LPN	14	2.08
CLC, SLP	20	2.97
CLC, MD	9	1.34
CLC, OT	7	1.04
CLC, PT	1	0.15
CLC, DO	1	0.15
CLC, IBCLC	47	6.97
Other	138	20.47

This table contains respondents’ demographic data collected. CLC, certified lactation counselor; CLC, DO, certified lactation counselor, doctor of osteopathy; CLC, IBCLC, certified lactation consultant, international board-certified lactation consultant; CLC, LPN, certified lactation consultant, licensed practical nurse; CLC, MD, certified lactation consultant, medical doctor; CLC, OT, certified lactation consultant, occupational therapist; CLC, PT, certified lactation consultant, physical therapist; CLC, RN, certified lactation consultant, registered nurse; CLC, SLP, certified lactation consultant, speech and language pathologist; IBCLC, International Board-Certified Lactation Consultant; MD, medical doctor; PhD, doctor of philosophy.

This process was undertaken collectively by all authors. Human studies permission was granted by Curry College’s Institutional Review Board on May 6, 2022.

## 3. Results

The descriptive statistics showing the characteristics of the study sample are presented in Table 1. The sample consisted primarily of female-identifying respondents (*n* = 665, 98.6%) with a mean age range of 35–50 years (*n* = 310, 45.9%). Three quarters of respondents self-identified as White/Caucasian (*n* = 507, 75.2%) and 47.3% held a Bachelor’s Degree (*n* = 319). Less than half of respondents practiced lactation counseling and services in a city (*n* = 287, 42.6%). Workplace settings and credentials of respondents varied, with nearly half identifying as both a Registered Nurse (RN) and a CLC (*n* = 303, 44.9%) (Table 1). About half of the respondent’s place of work was in a hospital, specifically Postpartum Units (*n* = 159, 23.6%) and Labor and Delivery Units (*n* = 158, 23.4%) (Table 2). The geographic representation of the respondent sample was broad, with the largest cohort working in HHS Region 2 which includes Illinois, Indiana, Michigan, Minnesota, Ohio, and Wisconsin (*n* = 152, 22.5%) (Table 2).

**Table 2:** Characteristics of respondents’ workplace settings and geography.

	*N*	%
Workplace setting (*n* = 674)		
Hospital (labor and delivery)	158	23.44
Hospital (postpartum)	159	23.59
Hospital (NICU)	85	12.61
Hospital (other units)	53	7.86
Birth center	23	3.41
Family outpatient office/clinic	40	5.93
OB-GYN office/clinic	35	5.19
WIC office	129	19.14
Pediatric office/clinic	69	10.24
Private practice	76	11.28
Health department	61	9.05
Military healthcare	7	1.04
Community health center	24	3.56
Community support groups	31	4.60
Home visiting program	72	10.68
Head start/early intervention/healthy start	16	2.37
Other	63	9.35
Demographic Region (*n* = 674)		
HHS Region 1	61	9.05
HHS Region 2	89	13.20
HHS Region 3	48	7.12
HHS Region 4	121	17.95
HHS Region 5	152	22.55
HHS Region 6	50	7.42
HHS Region 7	41	6.08
HHS Region 8	72	10.68
HHS Region 9	28	4.15
HHS Region 10	12	1.78
Geographical workplace (*n* = 674)		
Rural	110	16.32
Town (less than 10,000 people)	73	10.83
City (10,000–250,000 people)	287	42.58
Large city (over 250,000 people)	135	20.03
Not currently practicing	69	10.24

This table contains the respondents’ workplace settings. NICU, neonatal intensive care unit; HHS, health and human services. HHS Region 1 Includes Massachusetts, Connecticut, Maine, New Hampshire, Rhode Island, Vermont. HHS Region 2 Includes New Jersey, New York, Puerto Rico, Virgin Islands. HHS Region 3 Includes Delaware, Washington D.C., Maryland, Pennsylvania, Virginia, West Virginia. HHS Region 4 Includes Alabama, Florida, Georgia, Kentucky, Mississippi, North Carolina, South Carolina, Tennessee. HHS Region 5 Includes Illinois, Indiana, Michigan, Minnesota, Ohio, Wisconsin. HHS Region 6 Includes Arkansas, Louisiana, New Mexico, Oklahoma, Texas. HHS Region 7 Includes Iowa, Kansas, Missouri, Nebraska. HHS Region 8 Includes Colorado, Montana, North Dakota, South Dakota, Utah, Wyoming. HHS Region 9 Includes Arizona, California, Hawaii, Nevada, American Samoa, Commonwealth of the Northern Mariana Islands, Federated States of Micronesia, Guam, Marshall Islands, Republic of Palau. HHS Region 10 Includes Alaska, Idaho, Oregon, Washington.

Survey respondents reported that during the initial phase of the pandemic, almost all of their workplaces implemented COVID-19 restrictions in an effort to stop the spread of the virus (*n* = 656, 97.3%). Respondents reported changes including disruption in access to care, which was exacerbated by staffing shortages along with insufficient and irregularly available appointments. The delivery of lactation support and services was also interrupted. Social distancing and mandatory stay-at-home orders, including the fear many patients felt during the initial outbreak phase of the pandemic, meant that some patients and clients did not go to their medical facilities, even for standard appointments (22). Respondents described how lactation visits and pediatric care visits were no longer prioritized during the early stages of the pandemic. Additionally, the insinuation of stigma and bias based on COVID-19 status in the care delivery system resulted in differences in how families with and without COVID-19 were treated, particularly in healthcare settings.

Respondents reported that healthcare decision making became based on fear and uncertainty rather than on formerly accepted evidence-based practices. Because social distancing and physical isolation were two of the main strategies utilized to curb the spread of the COVID-19 virus, the evidence-based practice of keeping mother and newborn together changed to an imposed separation (23). Participants indicated that they felt alone and in the dark during the initial outbreak phase of the pandemic, and felt unsupported in their professional roles. Many participants reported feeling unable to follow the rapidly changing slew of information being passed down from above in their healthcare facility.

The two analytic themes which emerged from the thematic synthesis were rescinding of evidence-based practices and the rescinding of access to lactation care.

## 4. Discussion

This study explored the experiences of a representative sample of CLCs during the initial outbreak of the COVID-19 pandemic, specifically in relation to how their roles changed and the challenges they faced working with all mothers, babies and families. To our knowledge, this is the first study focusing on the unique experiences of LSPs as a specialized member of the healthcare team, with a focus on the adaptions and changes these professionals were asked to make at the start of the pandemic. This study serves as an addition to the research literature regarding healthcare professionals and their experiences working in the field at this time (24–27).

Responses are quoted below in order to amplify the findings and elevate the unique experiences of LSPs. Respondents are differentiated by their assigned number (respondent #). To enable succinct reporting of relevant quotes, omitted words have been indicated by an ellipsis (…) and added text to provide context/ correction is indicated by words in brackets ([]).

### 4.1. Rescinding of evidence-based practices

Healthcare professionals rely on established, evidence-based practices and guidelines to provide care. Empirical evidence demonstrates that “the principal benefit of (clinical) guidelines is to improve the quality of care received by patients” (28). It has been widely reported that during the initial days of the pandemic, it was difficult to discern safe and ethically sound treatment options. One review of public health responses to COVID-19 in New York City, USA explored how there was no guidance at all for crisis standards in the metropolitan area, which led to uncertainty and fear among healthcare professionals (29). A review of professional society guidelines illustrates that many did not adhere to the World Health Organization (WHO) recommendations, which recommended that breastfeeding continue as a protective factor for mothers and infants, and other evidence-based practices, such as rooming-in and skin-to-skin contact between mother and infant be continued (30). The CDC was very influential during the pandemic in the U.S. They relayed consistent messaging to the general public on the management of COVID-19 and preventing infection, however, their guidance did not adhere to the WHO recommendations (15). These misaligned professional guidelines resulted in a public view that“the U.S. was inconsistent, ambiguous, or in explicit contradiction…in their recommendations throughout the outlined time period” (31–33). Respondents provided their experience of this lack of guidance as well as the incoherent administration of evidence-based practices in the early months of the pandemic.

“…Providers had little guidance.” (Respondent #415)

“There was a binder on our labor and delivery unit and all policy changes were contained for staff to read and refer to.” (Respondent #3)

“(There was) just a lot of different information coming in.” (Respondent #630)

“There was much discussion about what could be done to improve care but decisions were being implemented from the ‘top’. Nurses/lactation staff struggled to do what we could to provide the best care possible while avoiding contamination or spread of the virus.” (Respondent #38)

“There was no guidance or support, only restrictions.” (Respondent #345)

COVID-related changes were adapted quickly. Concepts were new and constantly changing. CLCs reported that the reason for new policies and procedures stemmed from the advice of organizations outside of the healthcare system.

Evidence-based practice acknowledges that the breastfeeding experience is most successful when adequate professional and social support is available to the breastfeeding mother (34). The provision of continuous support during labor and birth is an essential component of getting breastfeeding off to an optimal start (35). Upon reflection, healthcare policy makers could have embraced the precautionary principle during the initial phase of the COVID-19 pandemic related to breastfeeding counseling. The precautionary principle suggests that decision-makers should resist practice changes until proposed practice changes can be studied thoroughly. In this context, breastfeeding is the precautionary approach. Evidence has demonstrated the risks of not breastfeeding, but at the start of the pandemic, the risks of breastfeeding with COVID-19 were unknown. Therefore, breastfeeding as a public health practice should have been prioritized until evidence was found to dispute that claim. It has since been determined that the survival benefits of breastfeeding greatly outweigh the fatality rates of infants with COVID-19 (36).

The respondents point to the disruption of evidence-based labor and birth practices, including social or professional support, during the birthing process or for lactation care in the postpartum period for all mothers during the pandemic.

“Individuals during the beginning of the pandemic were not allowed to bring partners or children into postpartum visits with OBGYN providers in my area.” (Respondent #45)

“Absolutely no support groups, no in-person appointments.” (Respondent #419)

“…It was constant change, the hospital did allow mothers to EBF (exclusively breastfeed), but it was while the mom was masked, once the feeding was finished, the baby was kept 6 feet away from mothers. It was hard to support a breastfeeding mother, I can only imagine how the mother felt.” (Respondent #137)

“Mothers and infants were being separated and discouraged to breastfeed if COVID positive.” (Respondent #66)

Practice decisions were reportedly made based on fear and uncertainty rather than evidence. The feelings of fear and uncertainty at the beginning of the pandemic were widespread (37). Empirical research explains that “one of the central emotional responses during a pandemic is fear” (38). The experience of LSPs and all of the families they worked with was not unique. National polls conducted at the start of the pandemic revealed a sharp increase in fear and worries related to the virus (37). Changes to policies and procedures in LSP workplaces may have been implemented more so out of fear than on evidence.

“…A lot of moms and dads had fear of infecting (their) baby.” (Respondent #630)

“Breastfeeding was discouraged because of the fear of transmission.” (Respondent #353)

“At the time the science was very new, and I believe many providers were unsure and did advise not to breastfeed if the mother had COVID or was exposed to COVID.” (Respondent #44)

“My clients would come to me and would tell me that they really wanted to breastfeed but that the baby was either removed from their hospital room for fear of transmission or were given bottles of formula because they didn't want to risk transmission in the hospital.” (Respondent #336)

“Mothers being afraid to breastfeed and just, in general, (were) in fear.” (Respondent #659)

Lactation counselors report that they experienced concerning changes in evidence-based care and practices at the onset of the pandemic. These ranged from barriers to all patient interactions because of restrictions in access to care to the abrogation of established routines known to support breastfeeding and continued lactation. The rescinding of evidence-based practices affected lactation support providers’ ability to provide support to all families in the hospital and in the community.

### 4.2. Rescinding of access to care

Evidence-based practices to support breastfeeding include having ongoing access to lactation care and support (13). Respondents in the current study recounted changes in the way lactation care was provided during the initial outbreak of the pandemic. Due to reduced staffing, workplaces and/or governmental organizations began triaging all breastfeeding mothers based on the urgency of their lactation concern. The practice of triaging affected the availability of LSPs and breastfeeding support resources for those mothers. The experiences of other clinical staff working in healthcare settings during this time has been described similarly. For example, Chandy and colleagues’ meta-synthesis included 15 qualitative studies of the lived experiences of healthcare providers during COVID-19, which reported unexpected burdens at work and new challenges to face (4). Respondents in the current study reported comparable experiences.

“We were only allowed to help a mother/baby under certain urgent circumstances. Otherwise, we were not allowed to see them in person.” (Respondent #24)

“The days/hours that lactation support was provided was cut down to 4 hours every other day (it had been offered 8 hours every day).” (Respondent #208)

“Our agency’s policy restricted all in-person interactions.” (Respondent #655)

“Our lactation consultant at the hospital was furloughed, they considered her non-essential.” (Respondent #641)

Triaging families meant that, in many cases, individuals that were able to have an appointment with a LSP at the start of the pandemic had to meet a certain criteria of urgency, even though breastfeeding is recognized as a public health concern in most of the world, and the inability of all nursing individuals to meet and work with a LSP undermines the nation’s goals to improve and enhance infant nutrition. Similarly, participant responses reflect the systemic undervaluing of breastfeeding as a public health priority in emergency situations and daily maternal and child health care practices.

Standard procedures of care for a newborn and mother are established based on evidence-based outcomes from leading health organizations, including the AAP, CDC and World Health Organization (WHO). Postpartum visits in particular allow a mother the chance to evaluate her breastfeeding experience and to receive often much-needed encouragement and management strategies from the provider, such as their LSP, in order to continue breastfeeding. Standardized care was quickly rescinded in light of the pandemic, especially in the realm of maternal and infant care.

“Patients were not allowed to come back to the hospital for the 2 days weight and color and feeding assessment initially with COVID.” (Respondent #3)

“Women left the hospital earlier than pre-pandemic. They often were discharged after 24 hours. Thus, the support from lactation and nurses on the postpartum unit was not available. Formula was given to the majority of my patients as they left the hospital before their milk came in.” (Respondent #178)

“Patients would not seek treatment during the early period of the pandemic and I saw a lot of patients end up with more serious infections and other issues due to fear of coming to the hospital during that time. We also stopped a lot of outpatient treatment and assistance with breastfeeding and this was hard on our mothers who were struggling to breastfeed.” (Respondent #124)

“Patients were being discharged so quickly from the newborn nursery that it almost seemed like mom’s were being set up to fail as they didn’t have enough time for education in the hospital.” (Respondent #148)

“I had a mom book a consultation when she was still at the hospital. It was really sad to hear from her that the hospital was short staffed, that there was no CLC that could support her.” (Respondent #559)

Utilization of essential health services tends to decline when a pandemic occurs (39). The current study demonstrates how standard postpartum visits were hurriedly scaled back, and how many mothers feared going into healthcare settings with their newborn infant due to the possibility of COVID-19 transmission. The fear observed through the lens of CLCs in the current study demonstrates the mechanism in which access to care was rolled back.

While CLCs and other healthcare professionals were unable to support breastfeeding parents at the start of the pandemic in the way they once did, the lack of support for the CLCs themselves was evident in the current study.

“Hospitals across the board were understaffed and in the midst of COVID and with PPE (Personal Protective Equipment), I saw a lack of support…” (Respondent #76)

“It was difficult when the DOH's (Department of Health) Community Health Worker home visiting program was de-funded since we relied heavily on them (some CLCs) to visit participants to observe breastfeeding & troubleshoot any bf (breastfeeding) issues-latch, positioning, etc.” (Respondent #159)

“Evidence was offered, but evidence does not appear to take into account social-emotional needs.” (Respondent #348)

“The biggest challenge was in the support provided by our hospital (there was no support for lactation during the pandemic) so many families came to me after weeks of struggling.” (Respondent #460)

“As an employee, I was never tested for COVID or asked if I had symptoms. When there was an outbreak on another unit that revealed 30% of their staff and many patients had a current infection we were changed to N-95 masks. Even with the outbreak, on our unit there was no testing.” (Respondent #392)

The current study reports that CLCs experienced changes to their own ability to access mental and physical healthcare at the start of the pandemic. This trend was seen for many healthcare providers, as explained in a recent meta-analysis and a recent qualitative systematic review of healthcare workers not feeling adequately supported during the pandemic (40, 41).

The U.S. has a history of troubling behavior towards patients based on virology status. The history of the human immunodeficiency virus (HIV) and acquired immunodeficiency syndrome (AIDS) illustrates how quickly those who are sick turn into the “other” (42). LSPs in the current study documented that breastfeeding was discouraged for those presenting with the virus, and how policies around birth and breastfeeding varied drastically between COVID-positive parents and COVID-negative parents.

“If pregnant women were COVID positive they received different care then (those who tested) negative.” (Respondent #633)

“Initially lactating parents were discouraged from caring for and breastfeeding infants when (the) parent was COVID-Positive, especially in the hospital setting. Pumping to maintain supply was not often discussed or encouraged.” (Respondent #228)

“For COVID positive moms, at the beginning were told to not have any contact with (the) baby for 24 hours until (the) baby was tested for Covid. She could pump but not be close to (the) baby.” (Respondent #254)

“Initially they didn't allow COVID positive patients to breastfeed.” (Respondent #653)

“(Breastfeeding was) only discouraged for COVID positive mothers, but encouraged to pump and dump to establish supply for when recovered from COVID they can breastfeed.” (Respondent #638)

“We placed all babies of COVID 19 + mothers in the NICU and did not allow the mothers to breastfeed or give their expressed milk.” (Respondent #539)

When faced with the unknown of the pandemic, new procedures leaned towards separation, rather than connection, with severe consequences on breastfeeding rates. “In the setting of COVID-19, separation of mother–newborn dyads impact breastfeeding outcomes, with lower rates of breastfeeding both during hospitalization and at home following discharge compared with unseparated mothers and infants” (43). Evidence-based practices to support breastfeeding, pumping and rooming-in were discouraged, thus inhibiting fair and equal access to care for lactating individuals and their babies (13, 44).

## 5. Strengths and limitations

The demographic characteristics of the respondents indicate that a representative sample of CLCs across the U.S. and territories were included in the survey results. The research may have been limited by the methodology of an optional online survey in that it may not have collected a true representation of all CLCs working in the field at the start of the pandemic. Conversely, as an online survey, this method may have increased access to participation among respondents due to accessibility. Empirical evidence has demonstrated the vast differences in how the pandemic disproportionately affected communities of color, including in the context of breastfeeding. The current qualitative survey did not elicit responses related to racial discrimination, which is a limitation and weakness of the design of the survey and its language.

Self-report bias and recall bias are also potential limitations of the current study. Common self-report biases that may have been present include a lack of introspective ability, response bias and one’s individual interpretation of the research questions. Recall bias in the current study may have been present due to the length of time that has passed since the start of the COVID-19 pandemic, or due to memory issues of the respondents.

## 6. Conclusion

To our knowledge, this is the first study examining the experiences of LSPs in the U.S. at the beginning of the COVID-19 pandemic. This study elucidated CLC experiences during this challenging time, including their perception of the lack of support for themselves as professionals and lack of support for new parents who intended to breastfeed. This study reemphasized the overall lack of support of breastfeeding as a public health goal in the U.S.

The findings of the current study indicate that, in the early days of the COVID-19 pandemic in the U.S., public health goals for breastfeeding were subsumed as evidence-based practice and access to care for breastfeeding families was rescinded. The rescinding of evidence-based practices which support breastfeeding, and the limitations placed by the healthcare reactions on access to care, may result in healthcare consequences that are worse than the pandemic itself (45).

These findings have important considerations for healthcare planning. Consistent and considered emergency management responses, particularly as they relate to pandemics and other viral diseases, should protect and preserve existing evidence-based guidance, particularly in relation to evidence-based care and access to care for breastfeeding and human lactation.

Sharing information and open research about COVID-19 and breastfeeding and the impact the pandemic had on healthcare provider mental and physical health status is key to improving future public health responses in the maternal and child healthcare field (46). The early publication and sharing of knowledge of breastfeeding rates declining and access to lactation care and support being severely limited across the country may have allowed public health organizations and hospital networks to respond more effectively in the later phases of the COVID-19 pandemic, including reclaiming maintaining evidence-based practices and gaps in access to care.

## Data availability statement

The datasets analyzed for this study will be made available upon request to interested researchers.

## Ethics statement

The studies involving humans were approved by Curry College’s Institutional Review Board. The studies were conducted in accordance with the local legislation and institutional requirements. Written informed consent for participation in this study was provided by the participants.

## Author contributions

JG, EM, and KB contributed to conception and design of the study. EM organized the database and wrote the first draft of the manuscript. JG, EM, KB, and KC conducted the thematic analysis. All authors contributed to the article and approved the submitted version.

## Conflict of interest

KC and KB are employed by the Healthy Children Project, Inc. The Healthy Children Project Inc. is an academic, non-governmental organization that provides educational opportunities for lactation support providers. EM is employed by the Academy of Lactation Policy and Practice. The Academy of Lactation Policy and Practice is a certification body that provides the Certified Lactation Counselor (CLC) accredited certification program, the Advanced Lactation Consultant (ALC) professional certification program and the Advanced Nurse Lactation Consultant (ANLC) professional certification program.

The remaining authors declare that the research was conducted in the absence of any commercial or financial relationships that could be construed as a potential conflict of interest.

## Publisher’s note

All claims expressed in this article are solely those of the authors and do not necessarily represent those of their affiliated organizations, or those of the publisher, the editors and the reviewers. Any product that may be evaluated in this article, or claim that may be made by its manufacturer, is not guaranteed or endorsed by the publisher.
